# Alterations of Functional Connectivity in Stroke Patients With Basal Ganglia Damage and Cognitive Impairment

**DOI:** 10.3389/fneur.2020.00980

**Published:** 2020-09-10

**Authors:** Guanqun Yao, Jing Li, Sha Liu, Jiaojian Wang, Xiaohua Cao, Xinrong Li, Long Cheng, Huafu Chen, Yong Xu

**Affiliations:** ^1^Department of Psychiatry, First Hospital/First Clinical Medical College of Shanxi Medical University, Taiyuan, China; ^2^Shanxi Key Laboratory of Artificial Intelligence Assisted Diagnosis and Treatment for Mental Disorder, First Hospital of Shanxi Medical University, Taiyuan, China; ^3^The Clinical Hospital of Chengdu Brain Science Institute, MOE Key Lab for Neuroinformation, School of Life Science and Technology, University of Electronic Science and Technology of China, Chengdu, China; ^4^Center for Language and Brain, Shenzhen Institute of Neuroscience, Shenzhen, China; ^5^MDT Center for Cognitive Impairment and Sleep Disorders, First Hospital of Shanxi Medical University, Taiyuan, China

**Keywords:** basal ganglia damage, cognitive function, degree centrality (DC), functional magnetic resonance imaging (fMRI), stroke, voxel-mirrored homotopic connectivity (VMHC)

## Abstract

**Background:** Stroke with basal ganglia damage (SBG) is a neurological disorder characterized by cognitive impairment. The neurobiological mechanism of cognitive impairment in stroke patients with basal ganglia damage (SBG patients) remains unclear. This study aimed to explore the underlying neurobiological mechanism of cognitive impairment in SBG patients using resting-state functional magnetic resonance imaging (rs-fMRI).

**Methods:** The differences in functional connectivity (FC) between 14 SBG patients (average age: 61.00 ± 7.45 years) and 21 healthy controls (HC) (average age: 60.67 ± 6.95 years) were examined using voxel-mirrored homotopic connectivity (VMHC) and degree centrality (DC). Moreover, we compared the cognitive functions of SBG patients with HC using the Chinese Revised Wechsler Adult Intelligence Scale (WAIS-RC) and Wechsler Memory Scale (WMS).

**Results:** Full-scale intelligence quotient (FIQ) (*t* = 2.810, *p* < 0.010) and memory quotient (MQ) (*t* = 2.920, *p* < 0.010) scores of SBG patients were significantly lower than those of HC. Compared with HC, significantly decreased VMHC values in the bilateral angular gyrus, supramarginal gyrus, inferior frontal gyrus, middle temporal gyrus, hippocampus, precuneus, precentral gyrus, and middle occipital gyrus and decreased DC values in the right supramarginal gyrus, bilateral angular gyrus, and right postcentral gyrus were observed in SBG patients. Moreover, the VMHC values in the angular gyrus, inferior frontal gyrus, supramarginal gyrus, and middle temporal gyrus and the DC values in the right supramarginal gyrus were significantly correlated with cognitive functions in all participants.

**Conclusion:** Our findings may provide a neural basis for cognitive impairments in SBG patients. Furthermore, local abnormalities of functional networks and interhemispheric interaction deficits may provide new ideas and insights for understanding and treating SBG patients' cognitive impairments.

## Introduction

Stroke, which induces complex chronic disability, is the third leading cause of death all around the world (1). Stroke disabilities include not only motor impairments but also cognitive dysfunctions. With common cognitive impairments of attention, memory, visuospatial ability, language, and executive function, stroke reduces the quality of life of patients, leads to high mortality, and increases the social burden (2). A previous study reported that a loss of blood vessel supply to the brain in stroke patients leads to structural damage of gray or white matter (3). The obstruction of the great vessels could directly result in ischemic necrosis of the brain functional units within the cerebral cortex (4). Previous studies of stroke suggested that the functional connectivity (FC) in the supplementary motor area, the inferior temporal gyrus, and the middle occipital gyrus differs from that of normal participants (5). Additionally, significant abnormalities were found in the cognitive networks of stroke patients, such as the default network (DMN), and the somatic motor network (SMN) (6). However, these studies were not based on specific stroke sites, which makes the study of the effects of specific sites on cognitive function in stroke patients particularly important.

The common sites of stroke include the basal ganglia region, which is rich in blood vessels. The basal ganglia region is involved in not only complex movements but also the regulation of advanced cognitive functions and non-motor complex behaviors that are concerned with processing and integrating different types of information (7). Thus, lesions of the basal ganglia result in complex functional impairments not constrained to just local functional abnormalities (8). Furthermore, basal ganglia damage may lead to disorders of other cortical areas and functional networks. However, basal ganglia damage has not received much research attention in terms of exploring the neuroanatomical basis of stroke with basal ganglia (SBG), and the potential neurobiological mechanism of post-stroke cognitive dysfunctions with basal ganglia damage remains unclear.

Resting-state functional magnetic resonance imaging (fMRI) is a non-invasive method to delineate the intrinsic functional organization and connectivity patterns in the brain (9), and it has been used to assess cognitive function (10) and explore the mechanism of different diseases (11). fMRI is an important tool for the study direction of the vascular injury mechanism of strokes that have a disordered signal of blood oxygen. Furthermore, the fMRI technique can reveal abnormal mechanisms in other cortical regions and functional networks caused by stroke (12). Voxel-mirrored homotopic connectivity (VMHC) is an indicator of the resting-state FC (rs-FC) strength between the two mirrored voxels in both hemispheres. The VMHC method has been widely used and has demonstrated the significance of interhemispheric coordination for human perception and performance (13). It has also been suggested that communication between the left and right hemispheres of the human brain is crucial for cognition and emotion processing (14). The VMHC has also been applied to study Parkinson's disease, schizophrenia, and insomnia (15). Additionally, previous VMHC analyses of stroke have shown that altered VMHC is associated with motor function assessment and illness duration (16). All of these studies demonstrated that interhemispheric rs-FC is important for delineating the physiological mechanism of brain diseases and identifying abnormal FC patterns, which could contribute to the disease-related cognitive dysfunctions of the brain in stroke patients with basal ganglia damage (SBG patients).

Graph theory has been utilized to study the complex network and characterize the functional organization of the brain (17). The degree centrality (DC), an index that mainly characterizes the importance of each node in the brain network, can measure the quantity of direct connections between a certain node and other regions (18). DC is an effective method to investigate abnormal function information in brain networks at the voxel level. DC has been used to study schizophrenia and ischemic stroke and identify new neural markers for these diseases (19). For example, with DC approaches, the previous studies revealed cortical–subcortical dissociation (20) and identified significantly lower communication and coordination of the DMN components in schizophrenia (21). The above evidence indicates that DC can provide novel insights into the global connectivity patterns and abnormal function in diseases to uncover their neural basis.

Given that few studies have focused on the association between the alterations of functional connection and comprehensive cognitive functional impairments in SBG patients, in our study, we focused on the effects of basal ganglia damage on other cortical regions and functional networks to reveal its associations with cognitive functions. VMHC and DC were utilized to comprehensively describe the voxel-wise FC patterns in SBG patients. Furthermore, the Chinese Revised Wechsler Adult Intelligence Scale (WAIS-RC) (22) and Wechsler Memory Scale (WMS) (23) were employed to assess subjects' cognitive functions. We hypothesized that local abnormalities of functional networks and defects of interhemispheric interaction may provide new ideas and insights for understanding and treating SBG patients' cognitive impairments.

## Materials and Methods

### Participants

Fourteen SBG patients (eight males; age bracket: 50–70 years, average age: 61.00 ± 7.45 years) were enrolled from the neurology department, and 21 healthy volunteers (HC) were recruited through advertising. The enrolled patients had mild motor impairment and no aphasia or other symptoms that would influence psychological scale assessment or MRI scanning. The National Institutes of Health Stroke Scale (NIHSS) was used to assess the neurological function impairment of SBG patients. This study was enrolled in the Chinese Clinical Trial Registry with registration number ChiCTR1900026358. The criteria for all patients were as follows: (1) stroke with basal ganglia damage diagnosed by a physician; (2) right-handedness before stroke; (3) 1 week to 3 months after stroke; (4) clear enough awareness to undergo cognitive evaluation. The exclusion criteria were as follows: (1) inability to perform cognitive evaluation or contraindication to MRI; (2) other serious disease that would affect the experiment; (3) any pharmacological therapy affecting functional connectivity, such as antiepileptics or antipsychotics. All subjects completed cognitive evaluation assessed by WAIS-RC and WMS and the rs-fMRI scan.

### Data Acquisition

A 3T Siemens MRI was used to scan all the participants. During MRI scanning, all the subjects were asked to close their eyes, relax, and think about nothing. Functional imaging was acquired by an echo planar imaging (EPI) sequence: flip angle = 90°, 212 volumes of every participant, 32 transverse slices, matrix = 64 × 64, voxel size = 3.75 × 3.75 × 4 mm, TR = 2,500 ms, TE = 30 ms. We also obtained the 3D T1-weighted images: flip angle = 9°, 160 transverse slices, voxel size = 0.9 × 0.9 × 1.2 mm, TR = 2,300 ms, TE = 2.95 ms, matrix = 240 × 256.

### Lesion Map

**Figure 1:**
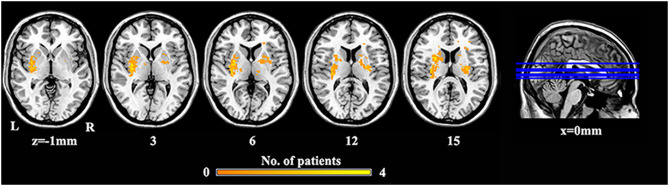
The lesion overlap map of SBG patients. Warm tones represent the superposition of the patient's lesion site. SBG patients, stroke patients with basal ganglia damage; L, left; R, right.

We performed a lesion imaging of SBG patients using T1 images (Figure 1). The lesion sites of each subject were carefully labeled using MRIcron. The anatomy and outline of the lesion can be clearly identified in the T1 images. Two neurologists labeled the stroke sites at T1 images by checking T1, T2, and diffusion-weighted imaging (DWI). We utilized the superposition of all patients' lesion masks to obtain the lesion mask of SBG patients after the spatial normalization.

### Data Preprocessing

Functional preprocessing was conducted using the SPM12 and DPARSF toolkits. The first 10 functional volumes were removed for the adaptation of subjects. The remaining 202 volumes were processed with slice timing and head movement correction. Any data whose maximum translation or rotation parameters exceeded ±2.5 mm or ±2.5° were excluded. The functional data were spatially normalized using the 3D T1 imaging, and the 3D T1 imaging was registered to mean functional data. We utilized 12-parameter nonlinear transformation to segment and normalize the 3D T1 imaging to Montreal Neurologic Institute (MNI) space. Additionally, we used a cost function modification to exclude the lesion areas and avoid deviation in the spatial normalization (24). We applied these transformation parameters to functional imaging. Furthermore, functional imaging was resliced with 2.0 mm^3^ and smoothed with a 6 × 6 × 6 full width at half maximum kernel. Finally, we processed functional data with the linear trends and temporal band-pass filtering (0.01–0.08 Hz). Additionally, the global signal, white matter signals, 24 motion parameters, and cerebrospinal fluid were regressed as covariates. Volumes of framewise displacement (FD) exceeding 0.5 mm were deemed motion outliers to further exclude the potential effect of head motion.

### VMHC Analysis

The VMHC index was calculated using the REST toolkit. For all the subjects, we computed the Pearson's correlation coefficient between each voxel's time series and its corresponding voxel time series in the symmetrical hemisphere. Furthermore, we applied Z transformation to the VMHC values to enhance the normality of data. The generated z-VMHC maps were used for subsequent statistical analyses (25).

### DC Analysis

Next, the DC index was analyzed. The DC was defined by calculating the correlation of a given voxel with each voxel of the whole brain. Weighted DC analysis was utilized to quantify the functional connectivity strength. Pearson's correlation coefficients (*r*) were computed between a given voxel and all other voxels in the rest of brain, and a threshold of *r* > 0.25, which can provide the most stable results, was applied to exclude weak connections caused by noise. FCs at the above threshold were averaged to obtain the DC value of the voxel. Furthermore, we applied Z transformation to the DC values to enhance the normality of the data. The generated z-DC maps were used for subsequent statistical analysis. Additionally, analyses of the other thresholds (*r* > 0.2 and *r* > 0.3) were performed to verify our findings.

### Statistical Analysis

We carried out independent-samples *t*-tests to calculate the differences in scale scores between SBG patients and HC. We also carried out two-sample *t*-tests using the SPM12 toolkit to analyze differences in VMHC between SBG patients and HC with gender, age, mean FD, and education level as covariates. The alterations of VMHC were identified at a statistical threshold of *p* < 0.01 and a minimum cluster size of 63 voxels (corrected with AlphaSim for multiple comparisons: voxel-wise *p* < 0.001 and lesions were excluded within the mask). The minimum cluster size was calculated by Monte Carlo simulation (1,000 iterations) using the REST AlphaSim program. We utilized REST to determine the peak coordinates of the significant clusters. The same procedure was used to identify the alterations of DC with a minimum cluster size of 46 voxels. Pearson's correlation analysis was performed to explore whether the associations of the significant VMHC and DC values were correlated with full-scale intelligence quotient (FIQ) and memory quotient (MQ) scores in all participants. The threshold of statistical significance was *p* < 0.05 with Bonferroni correction.

## Results

### Demographics and Cognitive Evaluation

Table 1 shows the demographics and cognitive evaluation of SBG patients and HC. Table 2 shows the demographic and clinical–radiological characteristics of each patient. No differences were found in age (Mann–Whitney *U* test, *p* = 0.967), gender (χ^2^ = 0.686, *p* = 0.407), education level (Mann–Whitney *U* test, *p* = 0.395), or mean FD (Mann–Whitney *U* test, *p* = 0.960). The FIQ (*t* = 2.810, *p* < 0.010) and MQ (*t* = 2.920, *p* < 0.010) scores of SBG patients were significantly lower than those of HC.

**Table 1:** Demographic and cognitive evaluation.

**Variable**	**SBG patients (*n* = 14)**	**HC (*n* = 21)**	***t*/χ^2^**	***p***
Ages (years)	61.00 ± 7.45	60.67 ± 6.95	145.5	0.967^a^
Gender (male/female)	8/6	9/12	0.686	0.407^b^
Education (years)	9.64 ± 3.37	10.29 ± 2.61	123.0	0.395^a^
Duration (days)	60.79 ± 15.48			
Lesion volume (cm^3^)	2.64 ± 2.47			
NIHSS	5.71 ± 2.16			
Mean FD	0.183 ± 0.151	0.164 ± 0.098	145.0	0.960^a^
FIQ	94.43 ± 13.78	108.10 ± 14.37	2.810	0.008^c^
MQ	100.70 ± 19.04	117.00 ± 14.13	2.920	0.006^c^

*HC, healthy controls; SBG patients, stroke patients with basal ganglia damage; MQ, memory quotient; FD, framewise displacement; FIQ, full-scale intelligence quotient; duration, the mean and range timing of MRI recording after stroke; NIHSS, National Institutes of Health Stroke Scale*.

a *Mann–Whitney U-test*.

b *Chi-squared test*.

c *Two-sample t-test*.

**Table 2:** The demographic and clinical–radiological characteristics for each patient.

**Numbers**	**Gender/Ages**	**NIHSS**	**Stroke type**	**Location of lesion**	**Lesion volume (cm^**3**^)**
1	Male/60	4	Ischemia	Lentiform nucleus	0.29
2	Female/69	9	Ischemia	Lentiform nucleus	4.27
3	Male/50	3	Ischemia	Right putamen	0.16
4	Male/65	5	Hemorrhage	Putamen	2.50
5	Female/70	8	Hemorrhage	Lentiform nucleus	3.79
6	Male/59	4	Ischemia	Caudate nucleus	1.08
7	Female/50	5	Hemorrhage	Left putamen	1.58
8	Male/50	6	Hemorrhage	Putamen	1.97
9	Male/65	3	Ischemia	Putamen	0.63
10	Female/69	7	Ischemia	Right putamen	3.58
11	Female/69	6	Ischemia	Right caudate nucleus	3.90
12	Female/55	4	Hemorrhage	Caudate nucleus	0.38
13	Male/59	10	Hemorrhage	Right putamen	9.52
14	Male/64	6	Ischemia	Putamen	3.25

*NIHSS, National Institutes of Health Stroke Scale*.

### Differences in VMHC

**Figure 2:**
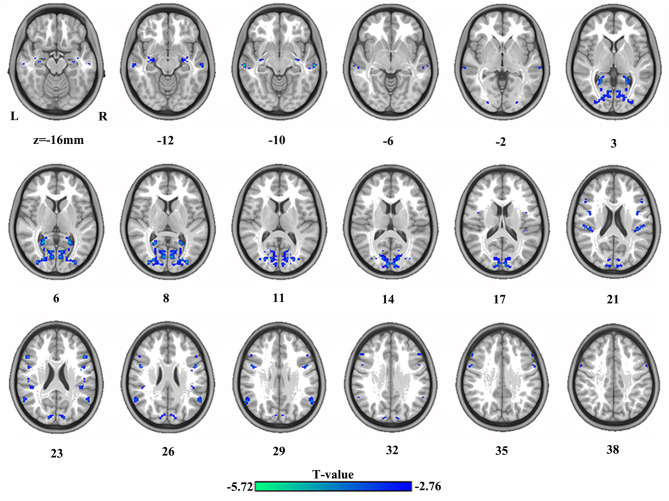
The differences in brain regions in VMHC between HC and SBG patients. The comparison between the two groups was acquired by a two-sample *t*-test (corrected with AlphaSim for multiple comparisons: voxel-wise *p* < 0.001 and lesions were excluded within the mask). Decreased VMHC values in SBG patients are represented by cool tones. SBG patients, stroke patients with basal ganglia damage; HC, healthy controls; VMHC, voxel-mirrored homotopic connectivity; L, left; R, right.

The differences in VMHC between HC and SBG patients are shown in Table 2 and Figure 2. SBG patients displayed decreased VMHC in the bilateral hippocampus, precuneus, angular gyrus, precentral gyrus, middle temporal cortex, middle occipital cortex, supramarginal gyrus, and inferior frontal gyrus.

### Differences in DC

**Figure 3:**
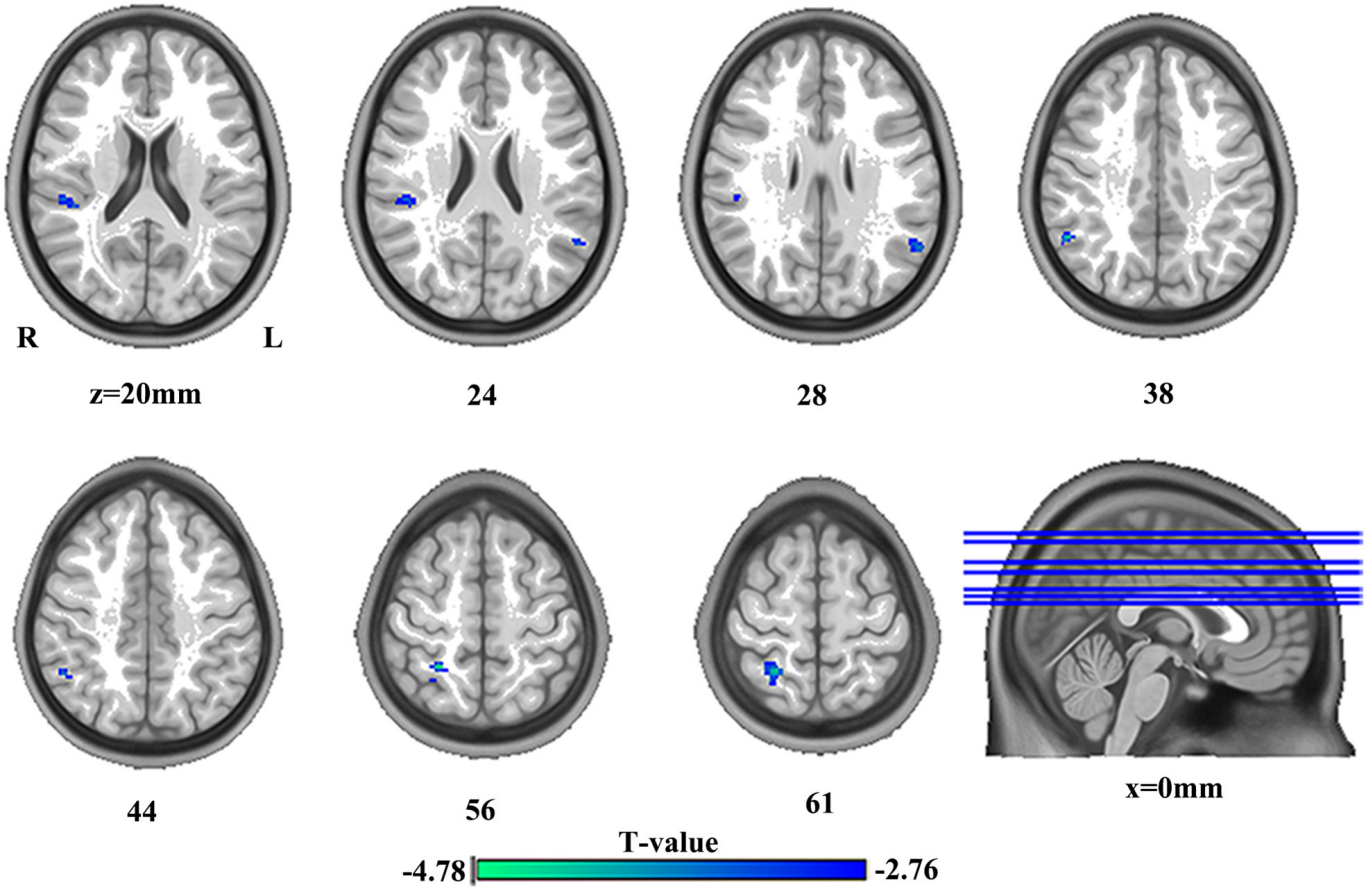
The differences in brain regions in DC between HC and SBG patients. The comparison between the two groups was obtained using a two-sample *t*-test (corrected with AlphaSim for multiple comparisons: *p* < 0.001). Decreased DC values in SBG patients are represented by cool tones. SBG patients, stroke patients with basal ganglia damage; HC, healthy controls; DC, degree centrality; L, left; R, right.

The significant group differences in DC between SBG patients and HC are shown in Table 3 and Figure 3. Compared with HC, we found that SBG patients displayed decreased DC in the right supramarginal gyrus, angular gyrus, and right postcentral gyrus. The analyses of additional thresholds (*r* > 0.2 and *r* > 0.3) showed similar findings, which are displayed in Supplementary Figure 1.

**Table 3:** The alterations of brain regions.

**Region**	**Cluster size (voxels)**	**MNI coordinates**			***T* value**
		***x*/mm**	***y*/mm**	***z*/mm**	
**VMHC**					
Hippocampus	64	±36	−8	−14	−3.92
Middle temporal gyrus	77	±64	−22	−8	−5.45
Middle occipital gyrus	608	±34	−88	8	−5.13
Precuneus	82	±24	−54	6	−5.72
Supramarginal gyrus	88	±50	−26	20	−4.09
Precentral gyrus	72	±50	4	26	−4.12
Angular gyrus	77	±58	−58	28	−4.20
Inferior frontal gyrus	64	±50	20	24	−4.87
**DC**					
Right angular gyrus	52	52	−52	38	−4.40
Right supramarginal gyrus	52	44	−34	20	−3.79
Left angular gyrus	53	−58	−58	28	−3.70
Right postcentral gyrus	82	26	−48	56	−4.78

*x, y, z, coordinates of primary peak locations in the Montreal Neurological Institute (MNI) space. VMHC, voxel-mirrored homotopic connectivity; DC, degree centrality*.

### Correlational Analysis

**Figure 4:**
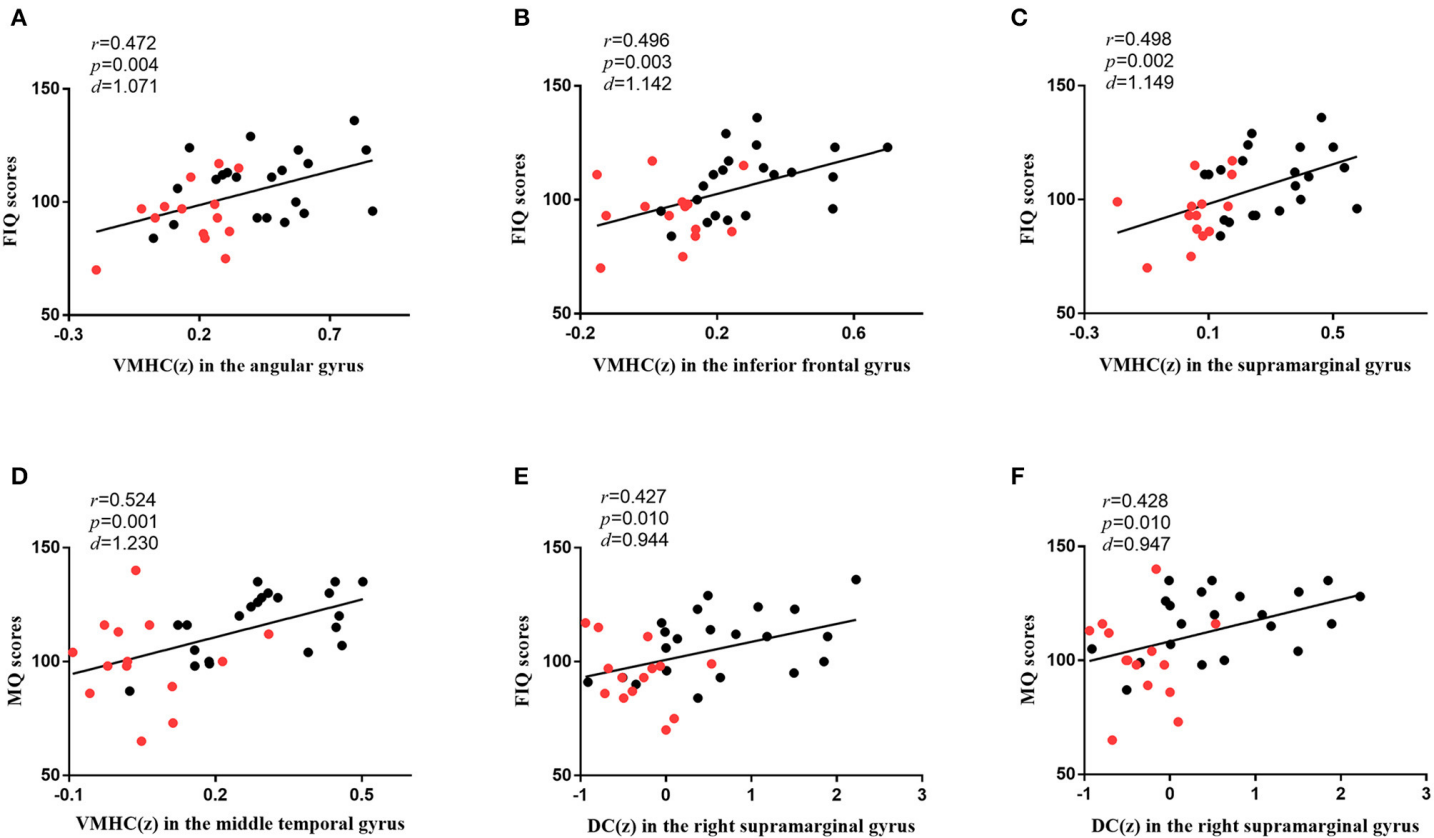
The correlations between brain functions and cognitive functions. **(A)** The diagram shows that the VMHC values in the angular gyrus were associated with FIQ scores (*r* = 0.472, *p* = 0.004, *d* = 1.071) in all participants. **(B)** The diagram shows that the VMHC values in the inferior frontal gyrus were associated with FIQ scores (*r* = 0.496, *p* = 0.003, *d* = 1.142) in all participants. **(C)** The diagram shows that the VMHC values in the supramarginal gyrus were associated with FIQ scores (*r* = 0.498, *p* = 0.002, *d* = 1.149) in all participants. **(D)** The diagram shows that the VMHC values in the middle temporal gyrus were associated with MQ scores (*r* = 0.524, *p* = 0.001, *d* = 1.230) in all participants. **(E)** The diagram shows that the DC values in the right supramarginal gyrus were associated with FIQ scores (*r* = 0.427, *p* = 0.010, *d* = 0.944) in all participants. **(F)** The diagram shows that the DC values in the right supramarginal gyrus were associated with MQ scores (*r* = 0.428, *p* = 0.010, *d* = 0.947) in all participants. Bonferroni correction with *p* < 0.05 was applied. *r* represents the Pearson correlation; *p* represents the significance level; *d* represents the effect size of the test. The red dots represent SBG patients, and the black dots represent HC. SBG patients, stroke patients with basal ganglia damage; HC, healthy controls; MQ, memory quotient; FIQ, full-scale intelligence quotient; VMHC, voxel-mirrored homotopic connectivity; DC, degree centrality.

As shown in Figure 4, in all participants, the VMHC values in the angular gyrus (*r* = 0.472, *p* = 0.004, *d* = 1.071), inferior frontal gyrus (*r* = 0.496, *p* = 0.003, *d* = 1.142), and supramarginal gyrus (*r* = 0.498, *p* = 0.002, *d* = 1.149) were correlated with FIQ scores, and those in the middle temporal gyrus (*r* = 0.524, *p* = 0.001, *d* = 1.230) were correlated with MQ scores. The DC values in the right supramarginal gyrus (*r* = 0.427, *p* = 0.010, *d* = 0.944) were correlated with FIQ scores, and those in the right supramarginal gyrus (*r* = 0.428, *p* = 0.010, *d* = 0.947) were correlated with MQ scores. Bonferroni correction *p* < 0.05 was applied. Other abnormal VMHC and DC values showed no significant correlations with FIQ or MQ scores.

## Discussion

In this study, we explored the neural basis of the alterations of cognitive function in SBG patients using VMHC and DC approaches. We found poorer intelligence and memory in SBG patients than HC. Meanwhile, the VMHC values and DC values in brain areas outside of the basal ganglia in SBG patients were lower than those in HC. Additionally, the VMHC and DC values were associated with cognitive functions. This study demonstrated that cognitive impairments caused by stroke with basal ganglia damage result not only from the site of direct ischemia or infarction but also from the functional abnormalities of distant functionally connected areas. These findings may contribute to the comprehension of cognitive impairments in SBG patients.

Accumulating evidence suggests that stroke may result in robust sensorimotor and cognitive deficits (26). A previous study combining static and dynamic analyses identified abnormal VMHC and dynamic VMHC in the precentral gyrus and other areas in stroke patients, and abnormal dynamic VMHC in the precentral gyrus showed a negative correlation with motor function (27). Our study focused on SBG patients and further identified the neural basis of cognitive impairments in stroke patients by combining VMHC and DC methods. The basal ganglia region is rich in blood vessels and is vulnerable to infarction or ischemic injury. Brain regions with large cognitive networks connected with the basal ganglia may experience functional impairment or compensation, known as functional recombination (28). Studying only motor dysfunction and cognitive function at stroke sites in stroke patients is insufficient to provide adequate information about the cognitive impairment of the whole brain. Here, the VMHC and DC were combined to explore the interhemispheric and global FC patterns to identify the possible neurophysiological basis of cognitive alterations in SBG patients. Our study demonstrated that the VMHC values of SBG patients were decreased in the hippocampus, precuneus, angular gyrus, precentral gyrus, and other areas, and DC values were decreased in the right supramarginal gyrus, angular gyrus, and postcentral gyrus compared with HC. These findings provide new information on how basal ganglia damage affects neural activities and interactions far from the lesion.

Our results suggested that the VMHC and DC values of the supramarginal gyrus and the VMHC values of the angular gyrus were positively correlated with cognitive function in all participants. The supramarginal gyrus is associated with language understanding and processing, and the angular gyrus, as a visual speech area, is related to a wide range of cognitive functions, such as attention, memory, and theory of mind (29–31). The supramarginal gyrus plays an important role in choosing appropriate actions in goal-oriented planning and acquisition and the use of tools (32). Additionally, in view of the role of the supramarginal gyrus in spatial processing and motor control, studies have shown that the decline of the supramarginal gyrus' function in stroke patients may be related to the decline of proprioception (33). We speculated that the functional damage of proprioception might be caused by the decrease of the functional connectivity of the supramarginal gyrus caused by basal ganglia damage. The angular gyrus is related to integrating semantic information and is the neurobiological basis of conceptual combination (34). A voxel-based morphometry (VBM) analysis of stroke showed that the reduction of angular gray matter volume might result in the decrease of semantic processing ability and speech orientation sensitivity (35). Therefore, the decrease of angular gyrus function might cause difficulties in understanding complex grammatical sentences and verbal working memory in SBG patients. Furthermore, the supramarginal gyrus and the angular gyrus are portions of the inferior parietal lobe (IPL). The IPL, which is the hub of multiple cognitive networks, is associated with a wide range of cognitive and behavioral functions, as well as bottom-up perception, attention, and non-directional thinking (36). Additionally, the IPL plays an important role in movement imagination and is related to the high-level regulation of the movement preparation stage. We speculated that dysfunction of the IPL may predict impairment of the ability to store and invoke motor information in SBG patients (37).

Our results further indicated that the VMHC values of the inferior frontal gyrus and the middle temporal gyrus were positively correlated with cognitive function in all participants. The inferior frontal gyrus is involved in the generation of creative cognition and contributes to the evaluation of original thinking. Additionally, the inferior frontal gyrus is considered to be a neural crossroads that distinguishes and processes information between abstract concepts and concrete concepts (38). The decrease of inferior frontal gyrus function may affect the information integration and transmission of higher cognitive functions in SBG patients. The middle temporal gyrus is an area associated with vocabulary storage that is functionally linked with the frontoparietal control system and increases neural activity in semantically demanding tasks (39). Previous lesion evidence showed that semantic and phonological fluency were affected by the inferior frontal gyrus and the middle temporal gyrus, respectively, in stroke patients (40). The decreased function of these regions may impair SBG patients' understanding and processing of speech and semantics.

Interestingly, these regions with changed VMHC and DC were mainly located within the DMN, the frontoparietal network (FPN), and SMN. The DMN, one of the most important brain networks, is related to cognitive functions such as episodic memory, self-projection, self-reflection, and mind-wandering thought (41). A study based on independent component analysis (ICA) showed that abnormal functional connectivity patterns exist in the DMN in stroke patients and are associated with cognitive impairments following stroke (42). Basal ganglia damage might lead to DMN dysfunction, which causes cognitive impairments in SBG patients. Meanwhile, the FPN, associated with executive function, is related to top-down cognitive control, particularly initiating and adjusting cognitive control (43). The SMN mainly includes the pre- and post-central gyrus and is related to sensorimotor ability. Additionally, some researchers have demonstrated that FCs within the SMN and the interaction between the FPN and the dorsal attention network (DAN) decrease significantly over time in Parkinson's patients, and the longitudinal decline in DAN-FPN interactions corresponds to increased cognitive impairments (44). Furthermore, a recent study on stroke showed that SMN–FPN connectivity is positively associated with the degree of motor and cognitive impairments (45). We speculated that basal ganglia damage might cause SMN–FPN dysfunction, and SBG patients might not be able to effectively combine movement and target information to perform organized and purposeful actions. All of these findings suggested that the DMN, FPN, and SMN are important for maintaining normal cognitive functions in SBG patients.

The present study has a few limitations worth noting. First, although we screened patients with basal ganglia lesions, stroke homogeneity might be a challenge. Second, the sample size was small, and further research should be performed with a larger sample size to verify the current findings. Third, cognitive function is so extensive that it can be measured with various methods. In future studies, we should focus on specific subdomains using comprehensive approaches. Fourth, combining task-based fMRI and multimodal neuroimaging may shed new light on the homotopic functional connectivity in SBG patients and related cognitive impairments in future studies. Additionally, VMHC analysis may not be suitable for subjects with apparent asymmetries in cortical structure. We will select participants with symmetrical cortical structure in future studies.

## Conclusion

This study was the first to explore the cognitive function of SBG patients using both VMHC and DC methods and identify the correlation between local functional areas and cognitive functions. Our findings provide preliminary evidence for the cognitive impairment of SBG patients. Local abnormalities of brain networks and disrupted interhemispheric interaction may provide new ideas and insights for the understanding and treatment of SBG patients' cognitive impairment.

## Data Availability Statement

The raw data supporting the conclusions of this article will be made available by the authors, without undue reservation.

## Ethics Statement

The studies involving human participants were reviewed and approved by the Ethics Committee of the Shanxi Medical University (Shanxi, China). The patients/participants provided their written informed consent to participate in this study.

## Author Contributions

YX devised and managed the research. GY and JL contributed to analyzing functional images and finished the paper. HC, XL, and JW focused on revising the manuscript. LC, SL, and XC recruited participants. All authors contributed to the article and approved the submitted version.

## Conflict of Interest

The authors declare that the research was conducted in the absence of any commercial or financial relationships that could be construed as a potential conflict of interest.
