# Supplementary material for: Alterations of Functional Connectivity in Stroke Patients With Basal Ganglia Damage and Cognitive Impairment
Source: Front Neurol. 2020 Sep 10;11:980. doi: 10.3389/fneur.2020.00980 (PMC7511868; doi:10.3389/fneur.2020.00980)
Supplement: Supplementary file 1 [file Data_Sheet_1.DOCX]

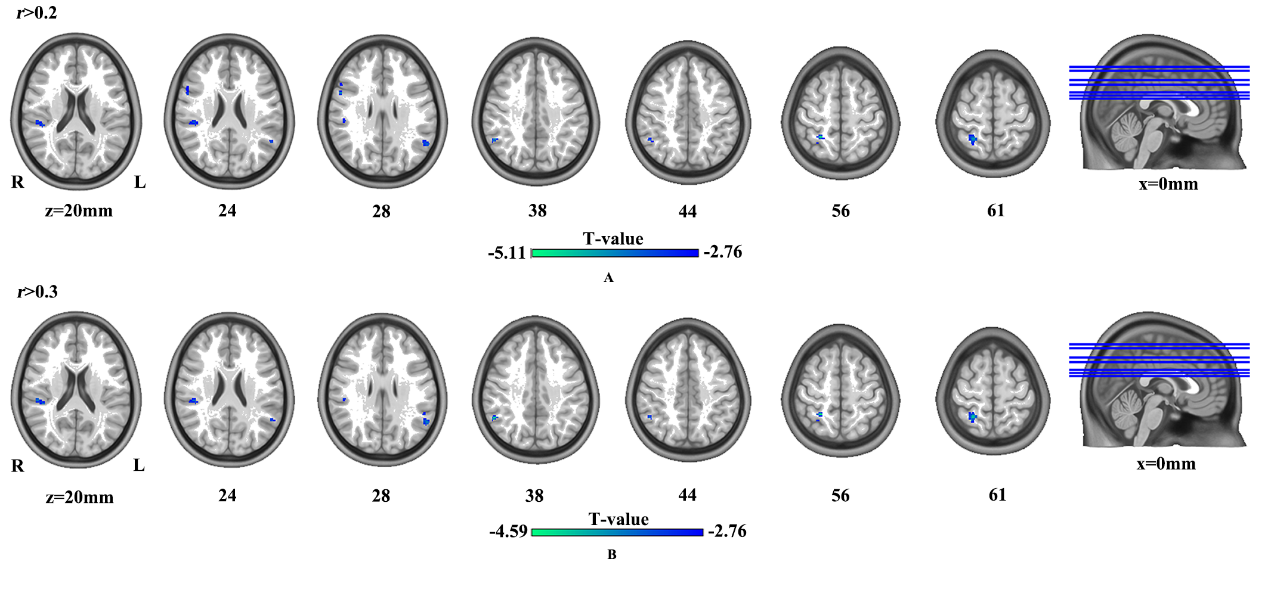


**Supplementary Figure 1. Reproducibility results in different *r* of DC between HC and SBG patients.** The analyses of additional threshold were chosen with *r*>0.2 (A) and *r*>0.3 (B). A: The alterations of DC were identified at a statistical threshold of *p*<0.01 and a minimum cluster size of 41 voxels (corrected with AlphaSim for multiple comparisons: voxel-wise *p*<0.001 and lesions were excluded within the mask). The minimum cluster size was calculated by Monte Carlo simulation (1000 iterations) using the REST AlphaSim program. B: The same procedure was used to compare the alterations of *r*>0.3 and the minimum cluster size is 50 voxels. Decreased DC values in SBG-patients were represented by the cool tone. Numbers below each axial slice refer to the z-plane coordinates of the MNI space, respectively. The results of the threshold of *r*>0.2 and *r*>0.3 were consistent with that of the threshold of *r*>0.25. SBG patients: stroke patients with basal ganglia damage; HC: healthy controls; DC: degree centrality; L: left; R: right.
